# Oxidized human serum albumin as a possible correlation factor for atherosclerosis in a rural Japanese population: the results of the Yakumo Study

**DOI:** 10.1186/s12199-017-0690-z

**Published:** 2018-01-02

**Authors:** Ryosuke Fujii, Jun Ueyama, Arisa Aoi, Naohiro Ichino, Keisuke Osakabe, Keiko Sugimoto, Koji Suzuki, Nobuyuki Hamajima, Kenji Wakai, Takaaki Kondo

**Affiliations:** 1 Department of Pathophysiological Laboratory Sciences, Nagoya University Graduate School of Medicine, 1-1-20 Daikominami, Higashi-ku, Nagoya, 461-8673 Japan; 2 Department of Clinical Physiology, Fujita Health University School of Health Sciences, 1-98 Dengakugakubo, Kutsukake-cho, Toyoake, 470-1192 Japan; 3 Department of Public Health, Fujita Health University School of Health Sciences, 1-98 Dengakugakubo, Kutsukake-cho, Toyoake, 470-1192 Japan; 4 Department of Health Administration, Nagoya University Graduate School of Medicine, 65 Tsurumai-cho, Showa-ku, Nagoya, 466-8550 Japan; 5 Department of Preventive Medicine, Nagoya University Graduate School of Medicine, 65 Tsurumai-cho, Showa-ku, Nagoya, 466-8550 Japan

**Keywords:** Human serum albumin, Redox state, Carotid intima-media thickness, Carotid plaque formation, Atherosclerosis, Oxidative stress

## Abstract

**Background:**

The effect of the redox state of human serum albumin (HSA) on the antioxidant properties of the entire body has been a focus of recent research. The usefulness of HSA redox state as a biomarker for reducing oxidative stress has been investigated in clinical settings; however, evidence for its significance as a health index in non-clinical settings is yet to be established. This study aimed to examine the associations between HSA redox state and the atherosclerotic indices of carotid intima-media thickness (IMT) and plaque formation in a rural Japanese population.

**Methods:**

We conducted a cross-sectional study as part of a health check-up program in the rural area of Hokkaido, Japan, at the end of August 2013. A total of 281 residents (124 men and 157 women) were included in the final analysis. Lifestyle-related data were obtained through a self-reported questionnaire, and ultrasound examinations were performed to measure IMT and determine plaque formation. The high-performance liquid chromatography postcolumn bromocresol green method was used to separate HSA into human nonmercaptalbumin and human mercaptalbumin (HMA).

**Results:**

We found a significant negative relationship between the fraction of HMA [f(HMA)] and IMT (standardized *β* = − 0.132, *p* = 0.03). Moreover, f(HMA) was significantly associated with plaque formation (*p* < 0.01) with an odds ratio of 0.89 (95% confidence interval, 0.81–0.97) for every 10% increment in f(HMA).

**Conclusions:**

We found that the HSA redox state, as determined by f(HMA), was associated with atherosclerotic indices in Japanese subjects. These results suggest that the HSA redox state indicates the risk of developing atherosclerosis.

## Background

Atherosclerosis is one of the major risk factors for cardiovascular diseases and cerebrovascular disorders. During the development of atherosclerosis, reactive oxygen species (ROS) induce low-density lipoprotein (LDL) cholesterol oxidation, vascular smooth muscle cell proliferation, endothelial cell injury, and inflammation [1]. Moreover, ROS have been implicated in the destabilization of atherosclerotic plaques by activating matrix metalloproteinase [2]. Therefore, the redox state is an important consideration for the prevention of atherosclerosis.

Human serum albumin (HSA), a simple protein comprising 585 amino acids with a molecular weight of 66 kDa, is produced in the liver and accounts for approximately 60% of plasma proteins. Of the 35 HSA cysteine residues, only N-terminal cysteine 34 (Cys-34) remains free. HSA exists in two main forms that are based on the state of Cys-34: human nonmercaptalbumin (HNA, oxidized form) and human mercaptalbumin (HMA, reduced form). HNA is subgrouped into two different states according to Cys-34 modification: HNA-1, which contains a disulfide that is reversibly oxidized by cysteine or glutathione, and HNA-2, which is highly oxidized to the sulfenic, sulfinic, or sulfonic acid form [3]. Although the fraction of each HSA form generally depends on age and pathological condition, studies have demonstrated that HMA, HNA-1, and HNA-2 account for 70–80%, 20–30%, and 2–5%, respectively, of the total albumin in a healthy young adult [4, 5].

HSA has many essential bioactive functions, including the transport of various endogenous and exogenous substances and the regulation of colloid osmotic pressure. In clinical settings, HSA has been used as a biomarker to evaluate the degree of hepatic and pancreatic disorders and nutritional status [6]. HSA is also known to be a predominant antioxidant against internal oxidative stress (OS). Some researchers reported that albumin can inhibit copper ion-dependent lipid peroxidation or OH generation and be a potent scavenger of ROS [7, 8]. Because the antioxidant capacity of HSA is primarily dependent on the modification of Cys-34, which is a main target site of OS to albumin, the ratio of the oxidized to the reduced form of HSA (HMA/HNA) may serve as a useful indicator for evaluating the systemic redox potential, reflecting the stage of disease progression or the effects of therapeutic interventions [9, 10].

Numerous clinical studies examined the relationship between the HSA redox state and the severity or prognosis of cardiovascular complications in patients with chronic kidney disease or those undergoing dialysis [11–14]. However, few epidemiological investigations have analyzed the association between HSA redox state and risk factors for lifestyle-related diseases in general populations. This study aimed to assess the redox state of albumin in a middle- and old-aged rural Japanese population and to examine its correlation with atherosclerotic risk as determined by ultrasonography.

## Methods

### Study subjects

We conducted a cross-sectional study as part of a health check-up program that was conducted in Yakumo Town, Hokkaido, in the northernmost region of Japan, at the end of August 2013. A self-administered questionnaire was distributed to the applicants before the check-up. Self-reported data were collected and missing answers were completed through interviews with municipal public health nurses at the screening site. The questionnaire was designed to collect data on the individuals’ medical history and lifestyle habits, including smoking status, alcohol consumption, and exercise habits. A total of 521 residents (220 men and 301 women) participated in the 3-day health check-up. The participation rate ranged from 0.7 to 5.0% based on the age groups (≤ 40, 40–49, 50–59, 60–69, 70–79, and ≥ 80 years old) of the residents listed in the Basic Resident Register.

During the check-up, fasting blood samples were collected through venipuncture and centrifuged within an hour of sampling. The serum specimens were stored at − 80 °C until the assay was performed. Routine biochemical analyses were performed in the laboratory of the Yakumo Town Hospital. Anthropometric measurements of body height and weight were obtained to calculate the body mass index (BMI, kg/m^2^).

Ultrasound examination was performed during the health check-up to measure the intima-media thickness (IMT) and determine plaque formation in carotid arteries. The results of the ultrasound examinations were reviewed mutually by two or three well-trained ultrasonographers after the health check-up to improve reliability; the details of this procedure have been described elsewhere [15]. We measured the maximum (max) IMT in both the right and left common carotid arteries (CCA), approximately 20 mm from the bifurcation, as recommended in the guidelines for the ultrasound evaluation of carotid artery lesions [16]. The mean value of the right and left max IMT was used for subsequent statistical analyses. The occurrence of plaques was defined as a focal raised lesion in either the right or left CCA, based on similar definitions used in earlier studies [15, 17, 18].

Individuals who took medications for the treatment of cerebrovascular disease, hypertension, ischemic heart disease, or diabetes mellitus were regarded as having a non-communicable disease. Due to a limited availability of serum specimens, we had to reduce the study population size. Therefore, we randomly selected the residential areas of the participants and eventually obtained a sample of 286 individuals (126 men and 160 women) so as to account for more than half of the 521 health check-up applicants. After excluding five individuals who did not undergo the ultrasound examination and biochemical analyses, the final analysis was conducted in 281 participants (124 men and 157 women).

### Measurement of HSA

HNA and HMA determination using high-performance liquid chromatography (HPLC) with an ultraviolet detector was previously reported by Sogami et al. [19]. In this study, we used the HPLC-postcolumn bromocresol green (BCG) method that was devised to prevent serum uric acid and bilirubin from interfering with chromatographic peaks [20]. The frozen serum samples were thawed at room temperature and filtered through Mini-UniPrep syringeless filters (Agilent, Tokyo, Japan). HPLC was performed to separate HMA and HNA, each of which was detected at a wavelength of 620 nm after reaction with the BCG reagent. The mobile phase reagent comprised N-methylpiperazine–HCl buffer (pH 4.5), 40 mM Na_2_SO_4_, and 3% ethanol. The BCG reagent comprised 150 mM citric acid, 3% Briji-35, and 0.3 mM BCG. We used distilled water that was deionized to 18 mΩ using the Millipore Milli-Q System (Millipore Co, Bedford, MA, USA) in all experiments.

The HPLC system used in this study was the Hitachi LaChrom Elite system (Hitachi, Tokyo, Japan) consisting of an isocratic pump (L-2130), an auto injector (L-2200), and a column oven (L-2300). The chromatogram was obtained using the Photodiode Array Detection system (Model L-2455). Shodex Asahipak GS-570 GS columns (100 × 75 mm ID) were used to separate HSA components before sample injection.

Because the HNA-2 peak was not adequately quantified in this experiment, its peak area was not considered in the subsequent analyses. To numerically evaluate the redox state of HSA based on the HPLC profiles, previous similar investigations used the fraction of HMA [f(HMA)] that signifies the proportion of the HMA peak area relative to the total HSA peak area [21–23]; therefore, we followed these reports.

### Statistical analysis

Data are presented as either means ± standard deviations for normally distributed variables or medians (1st–3rd quartile) for variables with a non-Gaussian distribution. After confirmation of a significant correlation between f(HMA) and max IMT, multivariate general linear regression was used to build a model using max IMT as the dependent variable. Moreover, multiple logistic regression analysis was performed to estimate the odds ratio (OR) and 95% confidence interval (CI) for the risk of IMT plaque formation associated with f(HMA) increments. In our statistical models, we adjusted for the following potential confounders: sex; age; high-density lipoprotein cholesterol, LDL cholesterol, triacylglycerides, and blood glucose levels; smoking history (never, ever, current); alcohol consumption (never, ever, current); exercise habit (almost none, 1–2 h/week, 3–4 h/week, ≥ 5 h/week); mean blood pressure calculated via the equation (2 × diastolic pressure + systolic pressure)/3; and history of medication intake for noncommunicable diseases. To evaluate the degree of multicolinearity between independent variables, the variance inflation factor (VIF) was calculated; no serious risk of multicolinearity was found (VIF < 4). The software used for the analyses was *R* ver3.3.1. All tests were two-tailed, and *p* values of < 0.05 were regarded statistically significant.

## Results

The basic characteristics of the subjects are summarized in Table 1. The average age of the study participants was 65.3 ± 9.2 years (range, 40–89 years). The means of f(HMA) and f(HNA) were 64.0 ± 6.8% and 36.0 ± 6.7%, respectively; these values are almost similar to those reported earlier [6]. Linear regression analysis without adjustment for confounders revealed a significant correlation between max IMT and f(HMA) [standardized regression coefficient (*β*) = − 0.25, *p* < 0.01]. After adjusting all confounders, a significant negative association remained between f(HMA) and max IMT (*β* = − 0.13, *p* = 0.03; Table 2). We then added an interaction term to examine whether the effect of f(HMA) varied by sex but found no significant differences in the effect (*p* = 0.33).

**Table 1:** Main characteristics of the subjects (*n* = 281)

Characteristics	Representative values^a^	
Age (years)	65.3 ± 9.2	
Body mass index (kg/m^2^)	24.2 ± 3.7	
Mean blood pressure (mmHg)^b^	90.5 ± 13.5	
Triacylglycerides (mmol/L)	0.96 (0.72–1.35)	
LDL cholesterol (mmol/L)	3.15 (2.56–3.62)	
HDL cholesterol (mmol/L)	1.55 (1.32–1.84)	
Blood glucose (mmol/L)	5.07 ± 0.85	
Max IMT (mm)^c^	0.90 (0.75–1.20)	
f(HMA) (%)^d^	64.0 ± 6.8	
	Frequency	%
Sex		
Male	124	44.1
Smoking status		
Never	151	53.7
Ever	96	34.2
Current	34	12.1
Alcohol consumption		
Never	163	58.0
Ever	13	4.6
Current	105	37.4
Exercise habits		
Almost none	146	52.0
1–2 h/week	68	24.2
3–4 h/week	32	11.4
≥ 5 h/week	35	12.5
Medication for non-communicable diseases		
Cerebral stroke	7	2.5
Hypertension	91	32.4
Angina	5	1.8
Diabetes	24	8.6

^a^Values are expressed as arithmetic means ± standard deviations or medians (25th–75th percentiles)

^b^Calculated as (2 × diastolic pressure + systolic pressure)/3

^c^IMT denotes intima-media thickness. Max IMT = (right carotid max IMT + left carotid max IMT)/2

^d^Fraction of human mercaptalbumin

**Table 2:** Linear regression analysis of the association between selected variables and max IMT

Independent variables		Unadjusted model		Age- and sex-adjusted model		Fully adjusted model	
		*β*_S_ (*β*_U_) [95% CI]	*p* value	*β*_S_ (*β*_U_) [95% CI^c^]	*p* value	*β*_S_ (*β*_U_) [95% CI^c^]	*p* value
f(HMA)^a^		− 0.25 (− 0.018) [− 0.027, − 0.010]	< 0.01	− 0.10 (− 0.007) [− 0.016, 0.001]	0.09	− 0.13 (− 0.010) [− 0.018, − 0.001]	0.03
Sex (male)		− 0.28 (− 0.277) [− 0.388, − 0.165]	< 0.01	− 0.24 (− 0.230) [− 0.340, − 0.130]	< 0.01	− 0.15 (− 0.151) [− 0.294, − 0.009]	0.04
Age		0.42 (0.023) [0.017, 0.028]	< 0.01	0.35 (0.019) [0.012, 0.025]	< 0.01	0.32 (0.017) [0.010, 0.025]	< 0.01
Body mass index		− 0.01 (− 0.002) [− 0.018, 0.014]	0.84			− 0.12 (− 0.016) [− 0.031, − 0.001]	0.04
Mean blood pressure^b^		0.26 (0.009) [0.005, 0.014]	< 0.01			0.13 (0.005) [0.000, 0.009]	0.03
Blood glucose		0.16 (0.005) [0.001, 0.009]	0.01			0.07 (0.002) [− 0.002, 0.006]	0.27
Triacylglycerides		0.01 (0.000) [− 0.001, 0.001]	0.82			− 0.02 (0.000) [− 0.001, 0.001]	0.68
HDL cholesterol		− 0.14 (− 0.005) [− 0.008, − 0.001]	0.02			− 0.02 (− 0.001) [− 0.005, 0.004]	0.80
LDL cholesterol		0.02 (0.000) [− 0.001, 0.002]	0.72			0.11 (0.002) [0.000, 0.003]	0.04
Smoking status	Ever	0.09 (0.097) [− 0.029, 0.223]	0.13			0.07 (0.075) [− 0.062, 0.211]	0.28
	Current	0.00 (− 0.006) [− 0.189, 0.177]	0.95			0.05 (0.074) [− 0.105, 0.253]	0.42
Alcohol consumption	Ever	0.14 (0.329) [0.054, 0.605]	0.02			0.10 (0.235) [− 0.020, 0.490]	0.07
	Current	0.11 (0.112) [− 0.007, 0.232]	0.07			0.03 (0.026) [− 0.100, 0.152]	0.69
Exercise habits	1–2 h/week	0.09 (0.098) [− 0.042, 0.239]	0.17			0.07 (0.082) [− 0.047, 0.211]	0.21
	3–4 h/week	0.07 (0.107) [− 0.080, 0.294]	0.26			0.05 (0.072) [− 0.096, 0.240]	0.40
	≥ 5 h/week	0.16 (0.238) [0.058, 0.419]	0.01			0.08 (0.118) [− 0.051, 0.287]	0.17
Medication for non-communicable diseases (present)		0.17 (0.176) [0.058, 0.295]	< 0.01			0.04 (0.043) [− 0.080, 0.165]	0.49

IMT denotes intima-media thickness, *β*_S_ denotes standardized *β*, *β*_U_ denotes unstandardized *β*, and CI denotes confidence interval

^a^Fraction of human mercaptalbumin

^b^Calculated as (2 × diastolic pressure + systolic pressure)/3

In the unadjusted logistic regression analysis, a significant relationship between f(HMA) and plaque formation was observed (OR = 0.87; 95% CI, 0.80–0.94; Table 3). A significant association between f(HMA) and the risk of plaque formation remained after full adjustment (OR = 0.89; 95% CI, 0.81–0.97) for every 10% increment in f(HMA).

**Table 3:** Logistic regression analysis for the association between selected variables and plaque formation

Independent variables		Unit	Unadjusted model		Age- and sex-adjusted model		Fully adjusted model	
			OR (95% CI)	*p* value	OR (95% CI)	*p* value	OR (95% CI)	*p* value
f(HMA)^a^		%	0.87 (0.80, 0.94)	< 0.01	0.92 (0.85, 1.01)	0.07	0.89 (0.81, 0.97)	< 0.01
Sex (male)			0.85 (0.76, 0.95)	< 0.01	0.87 (0.78, 0.97)	< 0.01	0.91 (0.79, 1.06)	0.23
Age		Year	1.14 (1.08, 1.21)	< 0.01	1.10 (1.03, 1.17)	< 0.01	1.07 (0.99, 1.15)	0.09
Body mass index		kg/m^2^	1.00 (0.86, 1.16)	0.98			0.88 (0.75, 1.04)	0.13
Mean blood pressure^b^		mmHg	1.05 (1.01, 1.09)	0.02			1.01 (0.97, 1.06)	0.58
Blood glucose		mmol/L	2.60 (1.37, 4.91)	< 0.01			2.10 (1.06, 4.14)	0.03
Triacylglycerides		mmol/L	1.10 (0.56, 2.15)	0.79			0.88 (0.44, 1.79)	0.73
HDL cholesterol		mmol/L	0.32 (0.08, 1.31)	0.12			0.91 (0.18, 14.8)	0.92
LDL cholesterol		mmol/L	0.87 (0.45, 1.71)	0.70			1.10 (0.56, 2.18)	0.79
Smoking status^c^	Ever		1.09 (0.97, 1.23)	0.15			1.03 (0.93, 1.19)	0.67
	Current		0.94 (0.79, 1.12)	0.48			0.96 (0.88, 1.16)	0.70
Alcohol consumption^c^	Ever		1.65 (1.27, 2.13)	< 0.01			1.59 (1.22, 2.07)	< 0.01
	Current		1.08 (0.96, 1.20)	0.20			1.01 (0.89, 1.15)	0.83
Exercise habits^d^	1–2 h/week		1.08 (0.95, 1.24)	0.25			1.07 (0.93, 1.22)	0.34
	3–4 h/week		1.07 (0.90, 1.28)	0.44			1.04 (0.88, 1.24)	0.62
	≥ 5 h/week		1.17 (0.98, 1.39)	0.08			1.10 (0.92, 1.30)	0.30
Medication for non-communicable diseases (present)			1.14 (1.02, 1.28)	0.02			1.02 (0.90, 1.16)	0.73

The number of participants with/without plaque formation was 90/191

OR denotes odds ratio. For continuous variables, the OR indicates the risk elevation per every 10-unit increment. CI denotes confidence interval

^a^Fraction of human mercaptalbumin

^b^Calculated as (2 × diastolic pressure + systolic pressure)/3

^c^“Never” was used as the reference

^d^“Almost none” was used as the reference

## Discussion

This study is the first to investigate the relationship between the redox state of HSA and indices of atherosclerosis in Japanese residents. We found an adverse association between f(HMA) with max IMT and a significant relationship between f(HMA) and the risk of plaque formation in the carotid artery.

HSA plays an essential role in mitigating the effects of OS on the human body; this function depends on the state of the major redox-sensitive site Cys-34 [8]. Several studies on the antioxidant mechanism of HSA demonstrated that HSA Cys-34 traps various ROS and reactive nitrogen species (RNS), including hydrogen peroxide, peroxynitrite, superoxide, and hypochlorous acid [24–26]. A recent review indicated that decreased HMA fractions ascribable to its oxidation by ROS or RNS causes the scavenging activity and antioxidant properties of HSA to decline [8]. Thus, the body might be more susceptible to OS and less capable of reduction in a state of diminished antioxidant potential. The observed relationship between low f(HMA) and atherosclerosis progression in this study may be attributable to this underlying mechanism. In a previous follow-up study, Terawaki et al. revealed that lower HMA conferred a considerable cardiovascular risk in patients undergoing peritoneal dialysis [27]. Furthermore, Hayashi et al. showed that, when compared to HSA, f(HMA) of human aqueous humor albumin was extremely low (66.4 vs. 3.5%, respectively) in 51 patients with senile cataract. While no age-matched cataract-free controls were included in their study, the authors postulated that a damaged antioxidant defense system in the aqueous humor contributed to cataract formation in these patients [28].

Previous studies have repeatedly emphasized on the unique properties of HSA as a major extracellular antioxidant; this suggests that the HSA redox state serves as an important biomarker for the overall potential of neutralizing OS. Although research interest in the HSA redox state has been long-standing [17], most studies have been conducted in clinical settings, with particular attention to patients with chronic kidney disease undergoing hemodialysis or cataract patients. In our investigation, we focused on the significance and applicability of the HSA redox state as a biomarker for cardiovascular risks in a community-dwelling Japanese population. Our results indicate that the HSA redox state can be a potential indicator of plaque formation risk and, to a lesser extent, of the progression of atherosclerosis in the carotid artery.

Several limitations should be considered when interpreting our results. First, several indices such as the max or mean IMT of the common carotid arteries (CCA-IMT) have been proposed for IMT evaluation [16]. CCA-IMT was validated as an indicator of generalized atherosclerosis in an autopsy analysis [29]. However, we employed max IMT in our statistical model because CCA-IMT was regarded as a complementary parameter whereas max IMT as a standard criterion. Second, the sample size of 281 in this study may not have been sufficient to maintain statistical power for the sex-specific analyses. Although the relationship between f(HMA) and max IMT did not seem to vary by sex according to the results of the linear regression model that incorporated an interaction term, sex-specific biology may have resulted in differential effects of OS on atherosclerosis. Third, because the participants who enrolled in the health check-up program were likely more health-conscious than ordinary residents, some selection bias may have affected our results. Fourth, the cross-sectional study design did not allow for causal conclusions on the pathways involved in the development of the atherosclerotic lesions when the antioxidant capacity of HSA was compromised. Therefore, the suggested usefulness of measuring the HSA redox state to predict longitudinal cardiovascular risks is tentative, although a previous report suggested that the measurements of the HSA redox state were an OS marker comparable to other established markers [12].

## Conclusions

The present study suggests that the HSA redox state is associated with atherosclerosis and might be a useful biomarker for atherosclerotic risk.

## Abbreviations

BCG: Bromocresol green
BMI: Body mass index
CCA: Common carotid artery
CI: Confidence interval
Cys-34: Cysteine 34
f(HMA): Fraction of human mercaptalbumin
HMA: Human mercaptalbumin
HNA: Human nonmercaptalbumin
HPLC: High-performance liquid chromatography
HSA: Human serum albumin
IMT: Intima-media thickness
LDL: Low-density lipoprotein
OS: Oxidative stress
RNS: Reactive nitrogen species
ROS: Reactive oxygen species
VIF: Variance inflation factor
